# Inhibition of experimental lung metastasis by systemic lentiviral delivery of kallistatin

**DOI:** 10.1186/1471-2407-10-245

**Published:** 2010-05-31

**Authors:** Ai-Li Shiau, Min-Li Teo, Shin-Yao Chen, Chrong-Reen Wang, Jeng-Long Hsieh, Meng-Ya Chang, Chih-Jui Chang, Julie Chao, Lee Chao, Chao-Liang Wu, Che-Hsin Lee

**Affiliations:** 1 Department of Microbiology and Immunology, National Cheng Kung University Medical College, Tainan, Taiwan; 2 Department of Biochemistry and Molecular Biology, National Cheng Kung University Medical College, Tainan, Taiwan; 3 Section of Rheumatology, Department of Internal Medicine, National Cheng Kung University Medical College, Tainan, Taiwan; 4 Department of Nursing, Chung Hwa University of Medical Technology, Tainan Hsien, Taiwan; 5 Graduate Institute of Clinical Medicine, Tzu Chi University, Hualien, Taiwan; 6 Department of Molecular Biology and Human Genetics, Tzu Chi University, Hualien, Taiwan; 7 Department of Biochemistry and Molecular Biology, Medical University of South Carolina, Charleston, USA; 8 Department of Microbiology, School of Medicine, China Medical University, Taichung, Taiwan

## Abstract

**Background:**

Angiogenesis plays an important role in the development and progression of tumors. Kallistatin exerts anti-angiogenic and anti-inflammatory activities that may be effective in inhibiting tumor metastasis. We investigated the antitumor effect of lentivirus-mediated *kallistatin *gene transfer in a syngeneic murine tumor model.

**Methods:**

Lentiviral vector encoding kallistatin (LV-Kallistatin) was constructed. The expression of kallistatin was verified by enzyme-linked immunosorbent assay (ELISA), and the bioactivity of kallistatin was determined by using cell proliferation, migration, and invasion assays. In addition, antitumor effects of LV-Kallistatin were evaluated by the intravenous injection of virus into tumor-bearing mice.

**Results:**

The conditioned medium from LV-Kallistatin-treated cells inhibited the migration and proliferation of endothelial cells. Meanwhile, it also reduced the migration and invasion of tumor cells. In the experimental lung metastatic model, tumor-bearing mice receiving LV-Kallistatin had lower tumor nodules and longer survival than those receiving control virus or saline. Moreover, the microvessel densities, the levels of vascular endothelial growth factor (VEGF), tumor necrosis factor (TNF)-α, and nuclear factor κB (NF-κB) transcriptional activity were reduced in the LV-Kallistatin-treated mice.

**Conclusion:**

Results of this study showed that systemic administration of lentiviral vectors encoding kallistatin inhibited the growth of metastatic tumor and prolonged the survival of tumor-bearing mice. These results suggest that gene therapy using lentiviruses carrying the *kallistatin *gene, which exerts anti-angiogenic and anti-inflammatory activities, represents a promising strategy for the treatment of lung cancer.

## Background

Kallistatin, a serine proteinase inhibitor, is first identified as a tissue kallikrein-binding protein, and has emerged as a novel inhibitor of angiogenesis. Kallistatin exerts a variety of biological effects in physiologic and pathologic responses, such as blood pressure regulation, inflammation and anti-angiogenesis [1-5]. It has been reported that kallistatin inhibited vascular endothelial growth factor (VEGF)-induced or basic fibroblast growth factor (bFGF)-induced the proliferation, migration and adhesion of endothelial cells and attenuated bFGF-induced capillary density in mice. Furthermore, a growing body of evidence indicates that chronic inflammation is considered to be one of the most important factors contributing to tumor development and progression. Most solid tumors contain many non-malignant cells, including immune and endothelial cells, which are important in inflammation. Inflammatory cells provide proteases that facilitate tumor invasion and matrix remodeling, accompanying with chemokines, growth factors, and angiogenic factors [6]. Kallistatin prevented inflammatory responses by reducing the accumulation of macrophages [7]. Based on these findings, kallistatin has the potential as a therapeutic agent for the treatment of tumor. We have recently reported adenoviral vector-mediated kallistatin expression ameliorated disease progression in the rat model of rheumatoid arthritis and osteoarthritis [2,8]. In this study, we exploited lentiviral vectors carrying *kallistatin *gene (LV-Kallistatin) as an antitumor agent in syngeneic murine tumor models. Via dual effect of anti-angiogenic and anti-inflammatory activities, LV-Kallistatin has the therapeutic potential for treatment of lung tumors.

## Methods

### Cell lines, plasmid, lentivirus and mice

Human rhabdomyosarcoma cells (TE671), 293T and mouse Lewis lung carcinoma (LL2) were cultured in Dulbecco's Modified Minimal Essential Medium (DMEM) supplemented with 10% cosmic calf serum (Hyclone, Logan, Utah, USA), 2 mM L-glutamine and 50 μg/ml gentamicin at 37°C in 5% CO_2_. Human umbilical vein endothelial cells (HUVEC) were cultured in EGM medium (Cambrex, East Rutherford, NJ, USA). The pcDNA3.1-hKallistatin plasmid was linearized with *Hind*III and filled in with Klenow fragment to form blunt end, and then the human kallistatin fragment was released with *Eco*RI from the linearized plasmid. The human kallistatin expression vector pWPXL-Kallistatin, under the control of elongation factor 1-α (EF-1α) promoter, was constructed by cloning the 1.3-kb cDNA fragment of human kallistatin into the *Pme*I/*Eco*RI sites of pWPXL. To produce recombinant lentiviruses encoding kallistatin or green fluorescence protein (GFP), 293T cells were transfected with pWPXL-Kallistatin or pWPXL, together with psPAX2 packaging vector and pMD2.G envelope vector by calcium phosphate precipitation, and the conditioned medium (CM) containing viral particles was harvested 48 h after transfection [9]. The titers of virus were determined on TE671 cells by fluorescence-activated cell sorter (FACS) analysis for GFP expression (BD Biosciences, San Jose, CA, USA). Briefly, approximately 2 × 10^5 ^TE671 cells were plated in six-well culture dishes and infected with serial 10-fold dilutions of viral vectors (10^-1 ^to 10^-5^) in serum free medium. Cells were collected 72 h after infection, and the number of GFP-positive cells was used to quantify the titer (transduction unit, TU).

Meanwhile, the titer of viral particles was performed using the Quick Titer lentivirus titer kit (Cell Biolabs, San Diego, CA, USA), which measures lentivirus-associated HIV p24 by enzyme-linked immunosorbent assay (ELISA). One ng/ml of lentiviral p24 corresponds to 1 × 10^5 ^TU/ml of the GFP-encoding lentiviral vector. The vector pWPXLLuc was derived from pWPXL where the GFP cDNA was replaced with firefly luciferase cDNA obtained from the pGL3-Basic vector (Promega, Madison, WI, USA). The EF1-α promoter was removed from pWPXLLuc at *Cla*I and *Swa*I sites and then replaced by the NF-κB responsive elements obtained from pGL2-IL8, resulting in pWPXL-NF-κBLuc [10]. The recombinant lentiviruses encoding luciferase were produced as previously described. For transduction of LL2 cells with the luciferase gene, cells were infected with recombinant lentiviruses carrying *luciferase *gene under the control of NF-κB responsive elements. As the lentiviral vectors did not contain selectable markers, luciferase-expressing stable LL2 clones were identified by monitoring luciferase expression in each isolated clone. LL2/NF-κBLuc clones with high level luciferase expression were used for further studies. Male C57BL/6 mice (6-8 weeks old) were obtained from the Laboratory Animal Center of the National Cheng Kung University. The experimental protocol adhered to the rules of the Animal Protection Act of Taiwan and was approved by the Laboratory Animal Care and Use Committee of the National Cheng Kung University. At indicated time points, mice were sacrificed, and the tissue lysates were assessed for luciferase activity with luciferase reporter gene assay system (Applied Biosystems, Foster City, CA, USA) using a luminometer (MiniLumat LB 9506; Berthold Technologies, Bad Wildbad, Germany).

### Cell migration, invasion and proliferation assay

The TE671 cells were infected with lentiviruses encoding GFP (LV-GFP) or LV-Kallistatin in different multiplicity of infection (MOI). The virus-containing medium from the infected cells was removed 8 h post-infection and fresh medium was added. The expression of kallistatin was verified by ELISA as described previously [11]. Forty-eight h after infection, the supernatant was collected for the migration and proliferation assays. Cell migration was assessed using a modified Boyden Chamber (Corning Costar, Cambridge, MA, USA). The membrane was coated with gelatin (100 ng/ml) for migration assay or collagen (100 ng/ml) for invasion assay. VEGF (10 ng/ml) or bFGF (15 ng/ml) was added at the lower compartment of the chamber. HUVEC (1 × 10^4 ^cells/well) or LL2 cells (1 × 10^4 ^cells/well) treated with CM were added to the upper compartment and incubated for 4 h. The filter was then fixed with 100% methanol for 8 minutes and stained by Giemsa stain solution for 1 h. Cells were counted randomly three images per well under a microscope. For the cell proliferation assay, cells were treated with serum-free medium for 24 h, and then incubated with 100 μl of CM from TE671 cells infected with lentiviruses in 10 MOI or mock infection in the presence of VEGF (10 ng/ml) or bFGF (15 ng/ml) for 48 h. Cell viability was measured with WST-1 assay (Dojindo Laboratories, Tokyo, Japan).

### Experimental lung metastatic models and Immunohistochemistry

To determine the expression of kallistatin after LV-Kallistatin injection, mice were inoculated with LL2 cells (5 × 10^5^) via the tail vein at day 0. LV-Kallistatin, LV-GFP (10^6 ^TU), or saline were injected into the mice via tail vein at day 15. The distribution of kallistatin in tissue was determined by ELISA at day 17. In the experimental lung metastatic model, mice were injected with LL2 cells (5 × 10^5^) via tail vein at day 0. Then the mice were treated with lentivirus (10^8 ^TU) by intravenous injection at day 1. To detect the protein and cytokine expressions, the lungs were collected at day 12. Levels of TNF-α and VEGF in the lung were determined by ELISA (R & D, Minneapolis, MN, USA). The protein content in each sample was determined by bicinchoninic acid (BCA) protein assay (Pierce Biotechnology, Rockford, IL, USA). Moreover, lungs from the tumor-bearing mice treated with lentivirus (10^8 ^TU) or saline were collected and weighted at day 25. To analyze infiltrating macrophages and microvessel density in the tumor sites, tumor-bearing mice were injected intravenously (i.v.) with LV-Kallistatin, LV-GFP (10^8 ^TU), or saline at day 1. The whole tumors were excised and snap frozen at day 20. Tumor angiogenesis and infiltrating macrophages were assessed by immunostaining as previously described [12].

### Statistical analysis

Statistical significance between groups was assessed by the unpaired Student's t-test. The survival analysis was performed using the Kaplan-Meier survival curve and the log-rank test. *p *< 0.05 were regarded as statistically significant.

## Results

### Expression of bioactive kallistatin via lentivirus-mediated gene transfer

**Figure 1:**
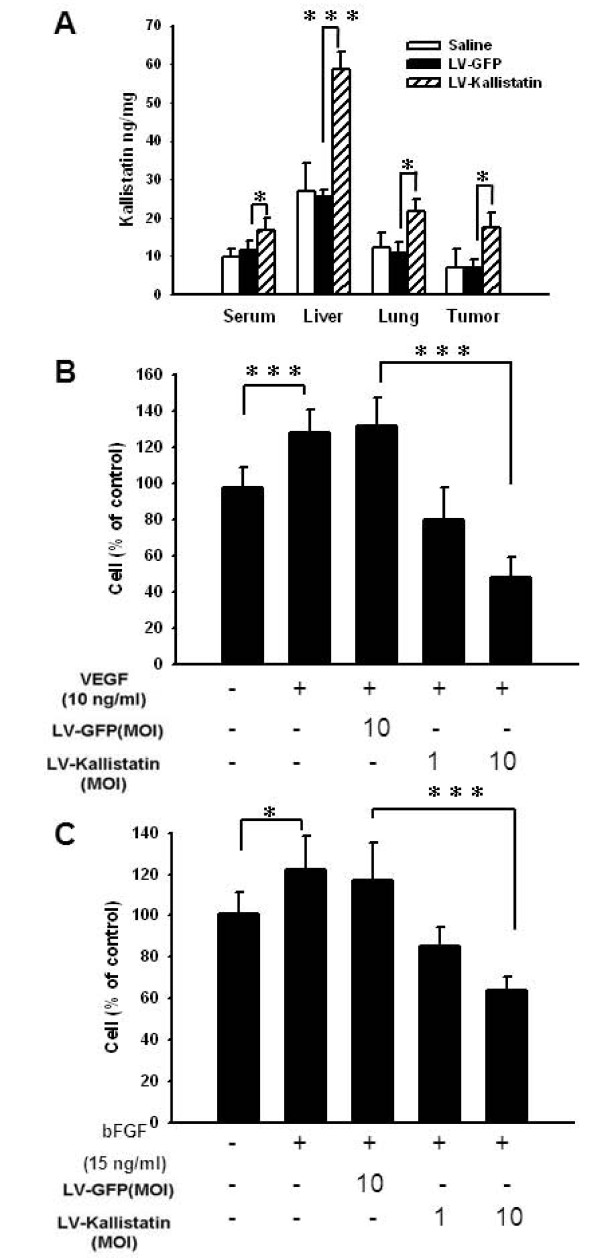
**Expression of bioactive kallistatin via lentivirus-mediated gene transfer**. (A) Groups of mice were inoculated with LL2 cells (5 × 10^5^) via tail vein at day 0. LV-Kallistatin or LV-GFP (10^6 ^TU), or saline were injected into tumor-bearing mice via tail vein at day 15. Mice were sacrificed at day 17, and tissues were harvested and measured by ELISA (mean ± SD, n = 3). The conditioned medium (CM) from LV-Kallistatin-infected TE671 cells inhibited (B) VEGF-induced and (C) bFGF-induced the proliferation of human umbilical vein endothelial cells (HUVEC). HUVEC (4 × 10^3^/well) were treated with serum-free medium for 24 h, and then incubated with 100 μl CM in the presence of VEGF (10 ng/ml) or bFGF (15 ng/ml) for 48 h. Cell viability was measured with WST-1 assay. (mean ± SD, n = 4, * *p *< 0.05,**p < 0.01, ***p < 0.001)

To test the feasibility of exploiting recombinant lentiviruses for gene delivery in cells, the expression of kallistatin was examined and characterized in TE671 cells infected with LV-Kallistatin or LV-GFP. TE671 cells transduced with lentiviral vectors were shown 1.3~2.1 times more efficiently transduction rate than 293T and HepG2 cells [13]. In this study, TE671 cells were used as the target cells to express the transgene. ELISA analysis was performed to examine the protein levels of kallistatin in the supernatant from TE671 cells. Kallistatin was detectable in LV-Kallistatin-treated cells, but not in LV-GFP- or saline-treated cells. To determine the gene expression in mice, we injected saline, LV-GFP, or LV-Kallistatin into tumor-bearing mice at day 15 and examined the expression of kallistatin at day 17. Low levels of signal in serum, lung, and tumor sites were detected in the saline-treated and LV-GFP-treated group. After LV-Kallistatin injection, the expression of human kallistatin was detected in all examined organs including tumor sites (Figure 1A). The CM from LV-Kallistatin-infected cells was tested for bioactive kallistatin. As shown in Figure 1, the CM from LV-Kallistatin-infected cells significantly reduced VEGF- (Figure 1B) and bFGF-(Figure 1C) induced the proliferation of endothelial cells. The inhibitive phenotype was in a dose-dependent manner. Together, these results showed that lentivirus-mediated gene transfer could produce biological active kallistatin.

### Kallistatin inhibits the proliferation and invasion of tumor cells

**Figure 2:**
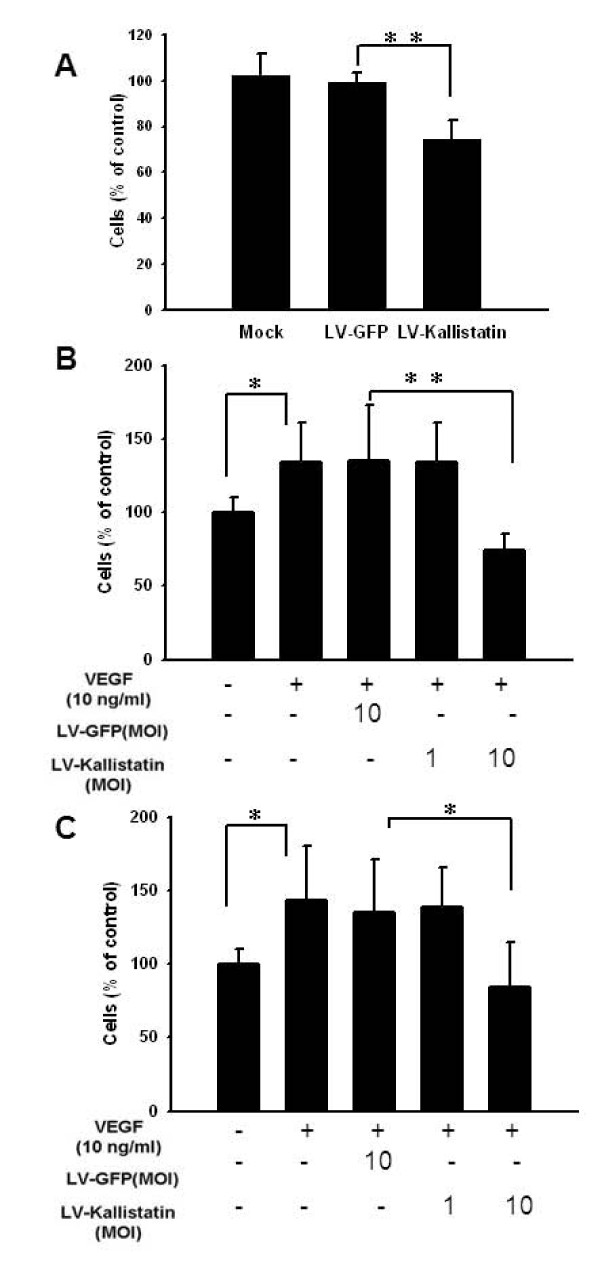
**Kallistatin reduced the proliferation, migration, and invasion of LL2 cells**. The CM derived from various-treated cells was diluted in serum-free medium at 1:1 and incubated with LL2 cells. (A) Cell viability was assessed by the WST-1 assay. The percentage of surviving cells (mean ± SD, n = 6) was calculated by comparing surviving cells of the CM-treated cells to saline-treated cells. Migration (B) and invasion (C) of LL2 cells (10^4^) were examined by using a 48-well Boyden chemotaxis chamber with membranes coated with gelatin (B) or collagen (C). (mean ± SD, n = 5, *p < 0.05, **p < 0.01)

Recombinant lentiviral vectors can transduce genes with great efficiency. Furthermore, systemic administration of lentiviruses resulted in the major transgene production in livers (Figure 1A) [14]. The results point out that host tissues can be transduced with lentiviral vectors and contribute to transgene production. We used the CM system *in vitro *to mimic the microenvironment *in vivo*. Figure 2A demonstrated that proliferation of LL2 cells was dramatically decreased upon addition of the CM from LV-Kallistatin-infected cells compared with that from LV-GFP-infected counterparts. Meanwhile, kallistatin in the CM dramatically reduced VEGF- or bFGF- induced LL2 cell migration and invasion (Figures 2B and 2C). In addition, the proliferation of LL2 cells was inhibited by LV-Kallistatin infection (Additional file 1). Taken together, these observations suggested that kallistatin inhibited the proliferation, migration, and invasion of tumor cells.

### Reduced metastatic nodules and prolonged survival in mice bearing experimental pulmonary metastatic tumors were obtained by systemic delivery of lentiviral vectors carrying kallistatin gene

**Figure 3:**
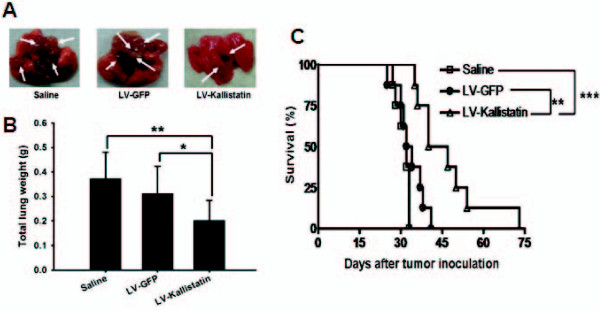
**LV-Kallistatin retarded tumor growth and enhanced the mice survival**. Groups of mice were inoculated with LL2 cells (5 × 10^5^) via tail vein at day 0. LV-Kallistatin or LV-GFP (10^8 ^TU), or saline were injected to mice via tail vein at day 1. Mice were sacrificed at day 25, and their lungs were photographed (A) and weighed (B) (mean ± SD, n = 8~9). Note that the numbers and sizes of tumor nodules indicated by arrows were reduced in mice treated with LV-Kallistatin compared with those treated with LV-GFP or saline group. (C) Kaplan-Meier survival curves of tumor-bearing mice with various treatments are shown. (n = 7~8). (*p < 0.05, **p < 0.01, ***p < 0.001)

Since inhibition of metastatic tumor is still a major challenge for tumor treatment, we next investigated whether LV-Kallistatin could inhibit pulmonary tumor nodules. The *in vivo *antitumor effects of LV-Kallistatin were evaluated in terms of tumor growth and survival in mice bearing metastatic pulmonary nodules. We injected mice with LL2 cells via tail vein at day 0, and then treated them i.v. with LV-Kallistatin, LV-GFP or saline at day 1 after tumor inoculation. Tumor-bearing mice were sacrificed at day 25 to determine the wet lung weight for quantifying the tumor burden in the lungs. As shown in Figures 3A and 3B, the tumor nodules and total lung weight were dramatically reduced in LV-Kallistatin-treated mice. The lung weight of mice treated with LV-Kallistatin was 35% less than that treated with LV-GFP or saline. Meanwhile, the treatment of LV-Kallistatin prolonged the survival of mice with pulmonary metastasis (Figure 3C). Results of this study showed that systemic delivery of LV-Kallistatin delayed tumor growth and prolonged mice survival.

### Inhibition of tumor angiogenesis by LV-Kallistatin

**Figure 4:**
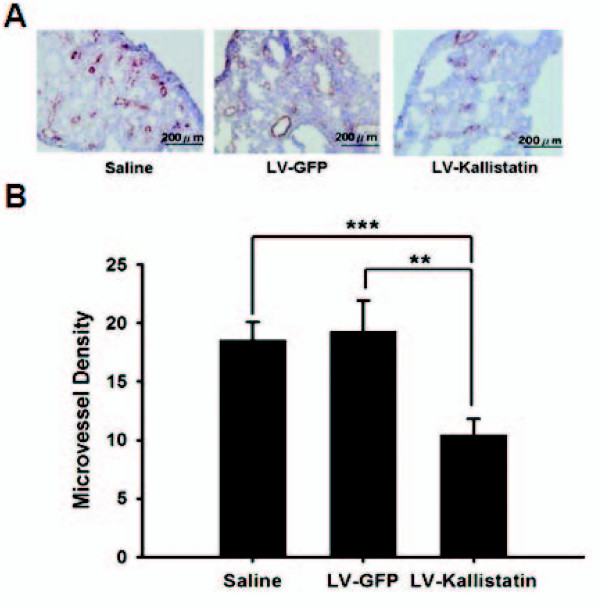
**Inhibition of angiogenesis in tumors by lentiviruses carrying *kallistatin *gene**. Groups of mice were inoculated with LL2 cells (5 × 10^5^) via tail vein at day 0. LV-Kallistatin or LV-GFP (10^8 ^TU), or saline were injected into mice via tail vein at day 1. (A) Lungs were excised at day 20, snap frozen and immunostaining with rabbit antibody against factor VIII-related antigen (×200). (B) Intratumoral microvessel density was determined by averaging the number of vessels in three areas of highest vessel density at × 200 magnification in each section. (mean ± SEM, n = 3 *p < 0.05, **p < 0.01, ***p < 0.001)

Microvessel density within the tumors was analyzed by immunohistochemistry at day 20. Tumors from LV-Kallistatin-treated mice revealed much less vessels than those from LV-GFP- or saline-treated counterparts, whereas no such difference was found between LV-GFP- and saline-treated groups (Figures 4A and 4B). Microvessel density in tumors receiving LV-Kallistatin reduced to 40% as compared with that in LV-GFP-injected group (19 ± 8 vessels/mm^2^, n = 10 versus 10 ± 4 vessels/mm^2^, n = 11) and in saline-injected group (19 ± 5 vessels/mm^2^, n = 11 versus 10 ± 4 vessels/mm^2^, n = 11). These results demonstrated that kallistatin gene delivery markedly suppressed the angiogenic response in the tumor sites.

### Effects of kallistatin on inflammation in tumor

**Figure 5:**
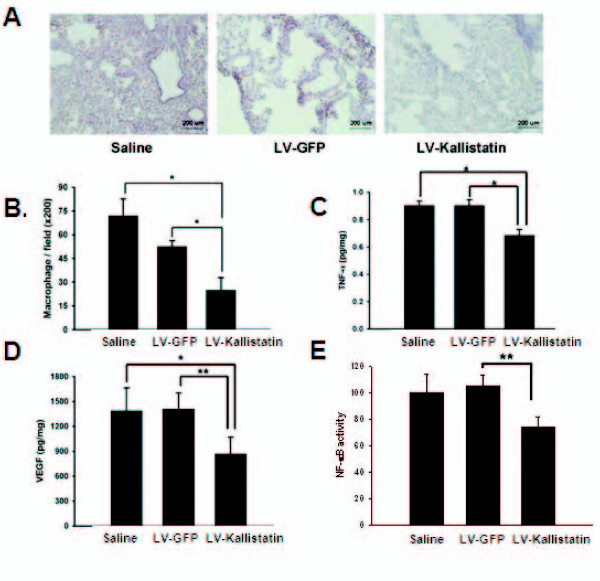
**LV-Kallistatin inhibited the inflammation in tumors**. Mice bearing LL2 or LL2/NF-κBLuc tumors at day 0 were injected with LV-GFP, LV-Kallistatin (10^8 ^TU) or with saline at day 1. (A) Tumors were excised at day 20, immunostained with antibodies against F4/80 (×200). (B) Macrophages were determined by averaging the cell numbers from three fields of highest positive-stained cell density at ×200 magnification in each section (mean ± SEM, n = 3~4). (*p < 0.05) Tumor-bearing mice were treated with LV-Kallistatin, LV-GFP (10^8 ^TU) or saline, and their lungs were collected at day 12. The levels of TNF-α (C), and VEGF (D) were measured by using ELISA. (E) The luciferase activities in tumors were measured at day 17. (mean ± SD n = 3~4, *p < 0.05, **p < 0.01).

Tumor progression depends, in part, on the density of tumor-associated macrophages (TAMs). There is a correlation between TAM abundance and poor prognosis [15]. TAMs are highlighted as not only a major participator but also an important regulator of inflammation. These macrophages actually promote the proliferation and metastasis of tumor cells by secreting a wide range of growth and pro-angiogenic factors, as well as pro-inflammatory cytokines [16]. In present study, the distribution of macrophages in the tumor sites was assayed by immunostaining. The numbers of infiltrating macrophages in mice treated with LV-Kallistatin were significantly decreased in comparison with those in mice treated with LV-GFP or saline (Figures 5A and 5B). As macrophages are major cytokine-secreting cells, we next examined the levels of TNF-α and VEGF in tumor microenvironment after different treatments. Tumors from LV-Kallistatin group contained lower concentrations of TNF-α and VEGF than those from control groups (Figures 5C and 5D). The presence of macrophages in solid tumors suggests that tumors grow at the sites of chronic inflammation. NF-κB signaling might play an important role in inflammation and tumor progression. To assess the activation of NF-κB signal transduction pathway, we performed a reporter assay using lentivirus-transduced LL2 cells containing the luciferase gene under the control of NF-κB responsive elements *in vivo*. LL2/NF-κBLuc cells were injected into the tail vein of mice, and the luciferase activity from lungs was determined. Figure 5E showed that luciferase gene expression was high, indicative of higher NF-κB activity, in the lungs of mice treated with saline or LV-GFP. Kallistatin inhibited the NF-κB transcriptional activity in tumor. These results suggest a potential role for kallistatin in the anti-inflammation and anti-angiogenesis, which may contribute to the inhibition of tumor growth.

## Discussion

Results of this study show that LV-Kallistatin inhibits the growth of orthotopic lung tumor via systemic administration. The expression of kallistatin in the tumor-bearing mice, which leads to decreased intratumoral microvessel density and chronic inflammation, may contribute to the antitumor effect of LV-Kallistatin on lung tumor. We demonstrated that intravenous administration of LV-kallistatin inhibited the tumor growth. However, complete tumor regression was not observed in the LV-Kallistatin-treated mice, and mice eventually died though the tumor growth was delayed. Different tumors may respond diversely to the angiogenic inhibitors [17,18]. Bergers *et al*. showed that various angiogenic inhibitors have distinct efficacy profiles depending on the stage of tumor development and probably the kinetics of cell growth [19].

Moreover, the anti-vector immunity is a potential issue for cancer gene therapy based on multiple systemic injections. The immunocompetent host previously exposed to the vector may cause their relative lack of efficacy. The lentiviral vectors, peudotyped with vesicular stomatitis virus glycoprotein (VSV-G), have been shown to be less sensitive to anti-vector neutralizing antibody, while displaying desirable characteristics, such as transduction of non-dividing cells, and long-term transgene expression [20,21]. However, in our previously studies, we injected adenovirus carrying *kallistatin *gene into knees of rats once per week for 3 weeks, and found the expression of transgene 10 days after the last virus administration. Our studies did not find any evidence of anti-kallistatin antibody in the treated animals [8]. A possible explanation for the limited transgene-triggered immune response is that the *kallistatin *gene is conserved in chimpanzee, dog, rat, and mouse [22,23]. The human kallistatin is very similar to mouse endogenous kallistatin. The anti-kallistatin neutralizing antibodies may not be produced dramatically during the short course of treatment. Accordingly, multiple injections of the recombinant lentiviruses are most likely to increase the kallistatin production, and enhance the antitumor activity in the short course of treatment.

Previously, human kallistatin levels were determined by ELISA in various human organs. The kidney had highest concentration of kallistatin, followed by liver, lung, prostate gland, and colon (34.8~130.5 ng/mg protein) among human tissues [24]. In our system, the tissue distribution of kallistatin in control mice was determined at higher concentrations in livers (27.0 ± 7.3 ng/mg), lungs (12.1 ± 3.9 ng/mg), and a lower concentrations in tumor sites (7.0 ± 5.0 ng/mg) by using human kallistatin ELISA system. Because we used polyclonal antibody against human kallistatin in ELISA system, the signals appeared in saline- and LV-GFP-treated mice were due to a cross-reaction. The expression of transgene in mouse tissues after systemic administration of LV-Kallistatin was determined by using human kallistatin ELISA system. The tissue distribution of mouse kallistatin remains to be investigated by using specific mouse kallistatin detective system.

Meanwhile, we found that kallistatin could inhibit the proliferation and migration of tumor cells. Indeed, it has been demonstrated that VEGF directly stimulates the growth of tumor cells [25]. Kallistatin inhibits the proliferation of endothelial cells and reduces production of paracrine factors, thus suppresses the proliferation of tumor cells. Our results found that VEGF could induce the migration and invasion of tumor cells (Figures 2B and 2C). Kallistatin may act by competing with VEGF and binding to heparin-sulfate proteoglycans [5]. Kallistatin suppresses VEGF-binding activity and angiogenic signaling cascades induced by VEGF. The anti-angiogenic effect of kallistatin may be similar to anti-VEGF antibody treatment pruning immature vessels in tumor sites. The vessel normalization and restoration of pressure gradients induced by VEGF blockade may explain the increased uptake of antitumor drugs and oxygen in tumor sites [26]. Furthermore, it has been reported that meloxicam augmented the antitumor activity of kallistatin [27]. Anti-angiogenic agents may contribute to improve the hypoxic condition of tumor sites by vascular normalization [28]. Hypoxia, a hallmark of many solid tumors, was reduced by angiogenic inhibitors [29]. Therefore, it was showed that kallistatin had the ability to reduce hypoxia inducible factor (HIF)-1 α expression [4], and may improve the hypoxic condition in the tumor microenvironment and increase the radiation or chemotherapy effects.

Several activities of kallistatin contribute to its antitumor effects. Kallistatin has ability to inhibit the inflammation and also reduce the intracellular superoxide formation [7]. Regulations of reactive oxygen species activity by kallistatin probably contribute to the anti-inflammatory activity. The effector cells involved in enhancing tumor growth appear to be macrophages, which are recruited to the tumor sites and produce TNF-α to stimulate tumor growth [30,31]. Our results found that kallistatin reduced the infiltrating macrophages, TNF-α production and NF-κB transcriptional activity in tumor sites. These findings suggest that kallistatin may affect the macrophages and provide a new insight regarding to the relation between inflammation and tumor. Therefore, it is plausible that the antitumor effect of LV-Kallistatin may be attributed not only to its effects on tumor endothelial cells, but also to its ability to down-regulate inflammation within the tumor sites. In agreement with previous reports, our results show that systemic administration of anti-angiogenic inhibitors to tumor-bearing animal results in down-regulation of VEGF expression in tumors [32,33]. In our studies, we did not observe any toxicity/side effects in animals, and the healthy lung did not show any toxic effect after LV-Kallistatin treatment. Therefore, combining our previous data with present ones, we suggest that lentivirus carrying *kallistatin *gene may be further explored as an anticancer agent for primary and metastatic tumors.

## Conclusion

We have demonstrated that *kallistatin *gene delivery can be achieved by systemic administration of lentiviral vectors carrying *kallistatin *gene and the gene expression can inhibit tumor growth and enhance survival in established metastatic murine lung tumor models. By taking advantages of the anti-angiogenic and anti-inflammatory effects of kallistatin, kallistatin gene therapy appears to hold promise for the treatment of solid tumors.

## Competing interests

The authors declare that they have no competing interests.

## Authors' contributions

ALS, CLW, CRW, JLH, MYC and, CJC designed the study. MLT and CHL did the experiments and drafted the manuscript. JC and LC provided the materials. All authors approved the final version of the manuscript.

## Pre-publication history

The pre-publication history for this paper can be accessed here:

http://www.biomedcentral.com/1471-2407/10/245/prepub

## Supplementary Material

Additional file 1 **LV-Kallistatin inhibited the proliferation of tumor cells**. LL2 cells (10^5^) were treated with saline, LV-GFP, or LV-kallistatin (10^6 ^TU) for 48 h. Cell viability was measured with WST-1 assay. The percentage of surviving cells was calculated by comparing surviving cells of the virue-treated cells to saline-treated cells. (mean ± SD, n = 4, **p < 0.01) Click here for file
