# Supplementary figures and images for: Inhibition of experimental lung metastasis by systemic lentiviral delivery of kallistatin
Source: BMC Cancer. 2010 May 31;10:245. doi: 10.1186/1471-2407-10-245 (PMC2893111; doi:10.1186/1471-2407-10-245)

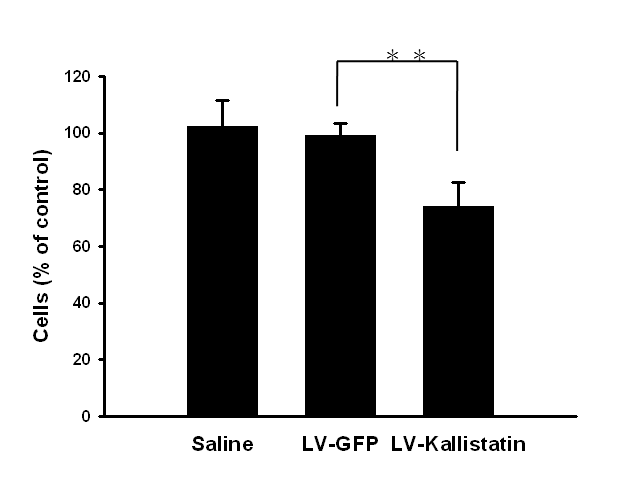

Supplement: Additional file 1 — LV-Kallistatin inhibited the proliferation of tumor cells. LL2 cells (105) were treated with saline, LV-GFP, or LV-kallistatin (106 TU) for 48 h. Cell viability was measured with WST-1 assay. The percentage of surviving cells was calculated by comparing surviving cells of the virue-treated cells to saline-treated cells. (mean ± SD, n = 4, **p < 0.01) [file 1471-2407-10-245-S1.TIFF]
